# Immunodiagnostic Detection of *Angiostrongylus cantonensis* Exposure on Hawaii Island Using Isogeographic 31-kDa Antigen

**DOI:** 10.4269/ajtmh.22-0643

**Published:** 2023-06-12

**Authors:** Susan I. Jarvi, Kirsten Nakayama, Praphathip Eamsobhana, Lisa Kaluna, Laura Shepherd, Yaeko Tagami

**Affiliations:** ^1^ Department of Pharmaceutical Sciences, Daniel K. Inouye College of Pharmacy, University of Hawaii at Hilo, Hilo, Hawaii;; ^2^ Department of Parasitology, Faculty of Medicine Siriraj Hospital, Mahidol University, Bangkok, Thailand

## Abstract

*Angiostrongylus cantonensis* is the leading cause of neuroangiostrongyliasis worldwide, and east Hawaii Island is a hotspot for the disease in the United States. A combination of glycoproteins with molecular weight of 31 kDa has been used as antigen to evaluate antibody response in human serum samples in Thailand with high specificity and sensitivity. In a previous pilot study, the Thailand-isolated 31-kDa proteins showed efficacy in dot-blot tests using serum samples from 435 human volunteers on Hawaii Island. However, we hypothesized that native antigen isolated from Hawaii *A. cantonensis* may exhibit higher specificity than the Thailand-isolated 31-kDa antigen due to potential minor variation in epitopes between isolates. In this study, 31-kDa glycoproteins were isolated by sodium dodecyl-sulfate polyacrylamide gel electrophoresis from adult *A. cantonensis* nematodes collected from rats captured on east Hawaii Island. The resultant proteins were purified by electroelution, pooled, bioanalyzed, and quantified. A subset of 148 samples from human participants of the original cohort of 435 was consented for this study, including 12 of the original 15 clinically diagnosed participants. Results of ELISA using the Hawaii-isolated 31-kDa antigen were compared with results of the same serum samples previously tested with both crude Hawaii antigen ELISA and Thailand 31-kDa antigen dot blot. This study shows a seroprevalence in the general population of East Hawaii Island of 25.0%, similar to previous findings of 23.8% seroprevalence in this cohort using crude antigen from Hawaii *A. cantonensis* and 26.5% using Thailand 31-kDa antigen.

## INTRODUCTION

The zoonotic nematode *Angiostrongylus cantonensis*, or rat lungworm, is the most common etiologic agent of eosinophilic meningitis worldwide.^1^^–^^3^ East Hawaii Island is a current hotspot of neuroangiostrongyliasis in the United States,^4^^–^^6^ with evidence of human disease in Hawaii having been recorded since 1958.^7^

The parasite has been identified in more than 30 countries worldwide,^8^^,^^9^ and human disease has been reported from at least 25 countries, with most cases originating in tropical or subtropical regions where the parasite is endemic.^8^ Isolated autochthnous human cases have arisen recently in the continental United States^10^^–^^12^ and in Europe,^13^ and surveys of rats and snails increasingly detect the presence of this parasite in previously undescribed geographic locations.^14^^–^^19^ Genetic variation among isolates has been described,^20^^–^^26^ and undescribed antigenic variation may also exist in different regions where the parasite’s life cycle has established.

The parasite can cause adverse health effects including, but not limited to, an eosinophilic meningitis known as neuroangiostrongyliasis, and infected individuals can exhibit long-term sequelae whether meningitis occurs.^27^^–^^30^ Ocular angiostrongyliasis can occur^31^ but visceral symptoms have not been classified formally as visceral larva migrans in this species, as has been suggested with *Angiostrongylus costaricensis*.^32^ Mortality is low, but morbidity can be severe and symptoms can persist for months or years, causing significant loss of vitality and having an impact on patients’ health as well as their economic viability.^30^^,^^33^^–^^36^ Although the CDC and other official sources of information about this disease have described a mild, self-limiting progression, presentation can be severe and even mimic multiple sclerosis^37^ due to demyelinization of nerves.^38^^–^^40^ Although rare, evidence also exists for growth of the parasite to adult stage in several cases in humans,^41^^–^^43^ and mating was found to have occurred in an adult female in the human lungs in one case but was not yet patent.^41^ It should be noted that the lungs and heart are not always examined for parasites in live patients or in autopsy.^44^ It has been suggested that human stools of patients in Hawaii should be screened for L1 *A. cantonensis*.^45^ Angiostrongyliasis has been a reportable disease in Hawaii since 2007 (Hawaii State Department of Health. Rat Lungworm Disease in Hawaii: Control and Prevention Legislative Report. March 2018; https://health.hawaii.gov/docd/files/2018/05/RLWD_2018_Leg_Report_032218.pdf). The disease is definitively diagnosed through physical detection of the parasite (which is rare), or the detection of parasite DNA in the cerebrospinal fluid (CSF) using a polymerase chain reaction (PCR) assay based on the first internal transcript spacing region (ITS1) gene region,^46^ or a more sensitive, recently developed quantitative PCR 3990 assay based on repeat regions.^47^ The ITS PCR assay has been available since January 2011^48^; it has been adopted by Hawaii’s Department of Health as the only definitive diagnostic in Hawaii and is the basis for officially reported cases, yet an overall detection rate of only 65.3% (32/49) was reported by this method in CSF (i.e., a 35% false-negative rate).^49^ The recently developed AcanR3990 assay has been shown to be much more sensitive and will likely be the assay of choice in the near future.^47^

Specific serological diagnosis of *A. cantonensis* has proved challenging. The 31-kDa proteins were first evaluated in 1997,^50^ and subsequently shown to have sensitivity and specificity of 100% for detection of antibodies against *A. cantonensis*.^51^ Serological analysis of CSF or serum was compared with real-time PCR and found that the immunodiagnosis was in agreement for 24 of the 26 patients.^49^ However, this study^49^ used crude antigen ELISA and Western blots (WB), not the isolated 31-kDa proteins. One of the two patients with discrepant results had a positive antibody test but was negative by PCR, described by the authors as a false-negative PCR result. The second patient was negative by PCR but had a positive antibody test that was attributed to failed antibody detection. A different study^52^ also reported discrepant specificity with the antigen but, again, used crude antigen (did not isolate the 31-kDa protein), and tested pooled serum. In yet another study,^53^ a 31-kDa protein was isolated, cloned, and expressed and subsequently was identified as the galectin-2 protein (rAcGal2). When tested by immunoblotting with human sera, sensitivity and specificity were 93.3% and 95.3%, respectively, indicating some degree of cross-reactivity.^53^ A rapid immunochromatic kit was developed based on rAcGal2, which showed sensitivity and specificity of 87.0% and 96.5%, respectively, again indicating some degree of cross-reactivity.^54^ The gel-isolated and purified 31-kDa antigen has been used most consistently in studies conducted in Thailand.^55^^–^^59^ In these Thailand trials, the glycoproteins comprising the purified 31-kDa antigen have shown 100% sensitivity and 98.72% specificity, and a rapid test kit has been patented.^59^^,^^60^

Antibody detection as early as 4 and 6 days post–symptom onset and as late as 67 days post–symptom onset has been reported based on WB data based on antibodies against the 31-kDa proteins.^49^ In the day 6 patient, PCR performed on CSF was negative, whereas the WB was positive, showing that serology may be more effective at certain time points in infection. Presence of DNA can be intermittent,^8^ whereas detection of antibodies may be possible long after seroconversion.

A presumptive diagnosis can be assigned by default when diagnostics fail, but symptoms, geographic risk factors, and exposure information combine to indicate potential infection with *A. cantonensis*.^8^ The cryptic nature of exposure (in regions such as Hawaii, where intentional ingestion of raw terrestrial mollusks is rare) and the difficulty in detecting early infection make this disease difficult to prevent, as well as to diagnose early enough to provide prophylaxis, although efforts to provide prophylactic treatment have been identified and implemented in some regions.^61^^,^^62^ An immunodiagnostic tool to identify antibodies to the parasite may help medical practitioners form a diagnosis when PCR is negative in suspected cases.

Although some still debate the possibility of cross-reactivity with the 31-kDa antigen, the main limitation of serological detection of *A. cantonensis* is the lack of availability of the native antigen needed for ELISA.^8^^,^^49^^,^^52^^,^^63^ To test whether isogeographic 31-kDa antigens provide superior detection of existing antibodies than the Thailand 31-kDa antigens, we performed indirect ELISA as previously described^64^ using 31-kDa proteins extracted from *A. cantonensis* worms isolated from rats on Hawaii island as an antigen source.

## MATERIALS AND METHODS

### Antigen preparation from adult worms.

Nematodes were collected in the course of previous animal studies.^4^^,^^65^ Nematodes were stored in 1X protease inhibitor (Biochem Cocktail set V EDTA-Free, Thermo Scientific, Waltham, MA) in 0.01 M PBS (Life Technologies, Grand Island, NY) at −80°C until crude antigen was prepared as described previously^64^ with the following modification: only female worms were used per Eamsobhana.^55^

### Protein isolation.

Crude antigen was prepared as described. The 31-kDa antigen from adult female worms was isolated from crude extract by sodium dodecyl-sulfate polyacrylamide gel electrophoresis (SDS-PAGE) as described previously^51^^,^^55^^,^^64^ with minor modification. Briefly, aliquots of ∼2.0 mL crude antigen extract were run on a series of 3-mm 12% acrylamide SDS-PAGE gels using a 16 × 20 cm Protean II xi Cell (Bio-Rad Laboratories, Hercules, CA) per manufacturer’s instructions. Hi-Mark^TM^ Pre-stained Protein Standard ladder was used to visualize the gel sections containing the 31-kDa proteins, and these sections of gel were excised manually. The gel slice was minced and then eluted with a Model 422 Electro-Eluter (Bio-Rad Laboratories) per manufacturer instructions. The resultant elute was filtered through Amicon Ultra-2 centrifugal devices (Millipore-Sigma, Burlington MA). Desalting/reducing was found to increase the amount of SDS in the elute, and this step of Eamsobhana’s protocols^51^ was discontinued after this result was discovered. After electroelution, proteins were quantified using High Sensitivity Protein 250 chips in an Agilent 2400 Bioanalyzer System (Agilent Technologies, Santa Clara, CA) according to manufacturer instructions. The quantified proteins were stored in 1.5 mL low protein binding microcentrifuge tubes (Eppendorf, Hauppauge, NY) at −80°C. The 31-kDa products of each gel were confirmed by bioanalysis and then pooled; the pooled antigen was reanalyzed by bioanalysis to confirm molecular weight of 31 kDa and to quantify the amount of protein in the pooled 31-kDa antigen preparations.

### Serum samples.

Serum samples were previously collected in 2015^64^ by the licensed staff of the Puna Community Medical Center and Clinical Laboratories of Hawaii. Samples were processed as previously described, deidentified of any personal data, and stored at −80°C. A total of 148 of the original cohort of 435 participants were reconsented for participation in the current study and their stored sera were used in this study.

### Controls.

The positive control sample was from an individual from east Hawaii who was clinically diagnosed by lumbar puncture in 2015; this sample was also chosen as a positive control in the pilot study^64^ due to the participant’s recent clinical diagnosis and the sample’s consistent and high mean absorbance. Similarly, the negative control sample used in the previous pilot study exhibited consistently low mean absorbance, was from an individual who visited Hawaii from the continental United States (Arizona), and reported no potential exposure to *A. cantonensis* and also served as a negative control in this study.

### ELISA.

Indirect ELISA was based on the methods described^64^ with the following modifications: Each plate included one positive control, one negative control, and multiple carbonate buffer controls (without antigen). After checkerboard titration to optimize antigen concentration, the isolated and purified 31-kDa *A. cantonensis* antigen was diluted with 0.05 M carbonate buffer pH 9.6 to a final concentration of 0.25 μg/well. ELISA plates were run on a BioTek 405 Select TS microplate washer (BioTek US, Winooski, VT) as described. Primary antibody (human serum) was diluted 1:200 in 1× phosphate-buffered saline (PBS; Life Technologies). Secondary antibody, goat polyclonal horseradish peroxidase–conjugated human IgG-Fc Fragment Antibody (Bethyl Laboratories, Montgomery TX) was diluted 1:1000 in 1× PBS. All samples were run in triplicate. Optical density (OD) was quantified using an iMark^TM^ Microplate Absorbance Reader (Bio-Rad Laboratories). The % ELISA value (EV) was calculated as previously described.^64^ Briefly, [(Sample OD − Negative control OD) / (Positive control OD − Negative Control OD)] × 100. To compare mean %EV between those participants diagnosed before and after 2014, a two-sample *t* test was performed in Minitab 18. The % coefficient of variation (CV) threshold was calculated as previously described.^64^ Briefly, the inter- and intra-assay mean absorbance of each sample in triplicate was set to 15% and 10%, respectively, and samples returning values outside %CV parameters were excluded from the study.

## RESULTS

### Isogeographic protein isolation.

**Figure 1.**
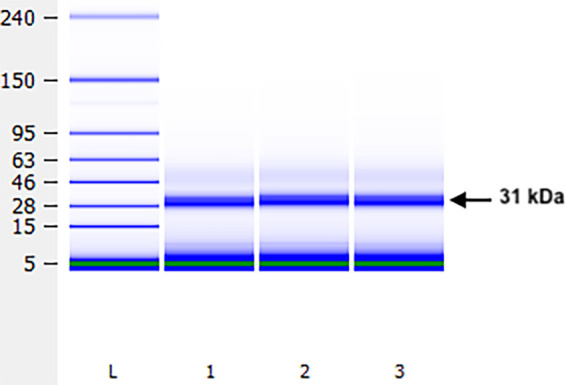
Crude antigen was prepared from 353 female *Angiostrongylus cantonensis*. Approximately 2% (2,537.36 μg) of the total preparation was recovered as isolated 31-kDa protein after electroelution. Bioanalysis of isolated antigen is visualized using an Agilent 2100 Bioanalyzer using the High Sensitivity Protein 250 Kit chip (L indicates ladder).

A total of 353 female *A. cantonensis* were used in the preparation of crude antigen for 31-kDa isolation. Approximately 2% of the volume of crude antigen used in each gel was recovered as isolated 31-kDa protein after electroelution. A total of 1,379 μL of purified antigen was estimated at 1.84 µg/µL, for a total estimated yield of 2,537.36 µg of 31-kDa isolate. An example of the bioanalysis of the isolated 31-kDa proteins is shown in Figure 1.

### Participants.

A total of 148 participants were reconsented from an original cohort of 435.^64^ In this previous study, the participants self-reported as either clinically diagnosed, positive (exposed or infected) or negative (uninfected and unexposed). These self-reported assignations and other demographic data points were retained for participants who were reconsented into this study. Twelve of the original 15 clinically diagnosed participants were reconsented in this study (Table 1). The %EVs of these participants of previous ELISA tests using crude antigen isolated from Hawaii *A. cantonensis* and the Thailand 31-kDa proteins by dot blot (either positive or negative) were compared with ELISA results using the Hawaii 31-kDa proteins as antigen (Table 1). Those that were dot-blot positive (*N* = 5) showed consistently higher ELISA %EVs than those dot-blot negative (*N* = 7) using either Hawaii crude antigen (%EV 67.0 versus 40.4), respectively, or the Hawaii-isolated 31-kDa antigen (%EV 67.0 versus 35.9), respectively.

**Table 1:** Self-reported demographics of clinically diagnosed participants

Residence	Gender	Age	When diagnosed	Where infected	Diagnosis procedure	Hospital admission	HI crude EV%	Thai 31 kDa dot blot	HI 31 kDa EV%
Pahoa	F	68	1981	Honokaa or Hilo	LP	HMC	18	P	17
Pahoa	F	63	2005	Kapoho (Puna)	Antibody test	NR	27	N	18
Pahoa	M	67	2007	Pahoa (Puna)	Eosinophil test	NR	60	P	61
Hilo	M	30	2008	Kapoho (Puna)	LP	HMC	53	N	77
Pahoa	F	12	2009	Pahoa (Puna)	LP	HMC	48	N	26
Pahoa	M	73	2009	Pahoa (Puna)	Presumptive	NR	16	N	14
Keaau	F	62	2010	Hawaii	Presumptive	NR	38	N	30
Hilo	F	39	2014	Hilo	LP/CT	HMC	100	P	100
Hilo	M	39	2014	Hilo	LP	HMC	70	P	72
Hilo	F	55	2014	Keaau (Puna)	Antibody test	NR	32	N	26
Pahoa	M	73	2015	Puna	Antibody test	HMC	87	P	85
Pahoa	F	59	2015	Puna	Presumptive	NR	69	N	60

CT = computed tomography scan; EV = ELISA value; F = female; HMC = Hilo Medical Center; LP = lumbar puncture; M = male; N = negative; NR = not recorded; P = positive. Included are previous results of %EVs using Hawaii crude antigen and dot-blot results using Thailand 31-kDa antigen^64^ and %EV using the Hawaii 31-kDa proteins isolated in this study.

### Demographics.

Date of birth and age were confirmed at reconsent; all other demographics were taken from the original data collected in the pilot study.^64^ The mean age of this cohort of participants (*N* = 148) at the time of sample collection was 59 years, with a range of 8 to 84 years. Female participants comprised 60% (*N* = 89) of participants and males, 40% (*N* = 59).

### Self-reported diagnosis.

In the previous study,^64^ participants were asked to self-report status as either clinically diagnosed with *A. cantonensis* infection, suspected infection or exposure (self-reporting positive), or no suspected infection/exposure (self-reporting negative). This previously collected data was used to categorize the participants in this study accordingly. Twelve clinically diagnosed participants (8%) self-reported clinical diagnosis (Tables 1 and 2); 21 (14%) participants self-reported as positive, or exposed and 115 (78%) self-reported no exposure or infection (Table 2).

**Table 2:** Summary of ELISA results with Hawaii 31-kDa antigen by self-reported diagnosis status

Diagnosis status	All participants	Clinically diagnosed	Self-diagnosed as positive	Self-diagnosed as negative
	*N* = 148	*N* = 12	*N* = 21	*N* = 115
Positive	13.5% (20)	33.3% (4)	23.8% (5)	9.6% (11)
Likely positive	11.5% (17)	16.6% (2)	14.3% (3)	10.4% (12)
Equivocal	34.5% (51)	41.7% (5)*	33.3% (7)	34% (39)
Negative	40.5% (60)	8.4% (1)†	28.6% (6)	46% (53)

EV = ELISA value. Samples with %EV > 70 are positive, %EV between 48 and 69.9 are likely positive, %EV 16 and 47.9 are equivocal, and %EV < 15.9 are negative. Number of participants in each column are represented as a percentage of the total and (number).

* One clinically diagnosed participant in this group was diagnosed after 2014; all others in this category reported diagnosis before 2014, with the earliest case diagnosed in 1981.

† This case was presumptively diagnosed in 2010.

### Indirect ELISA results.

Thresholds were consistent with the previously published study^64^: > 70%EV was determined to be a positive result; 48.0 to 69.9 was determined to be likely positive; 16.0 to 47.9 was determined equivocal; and values < 15.9 were determined to be negative.

ELISA results from this study are summarized in Table 2. Of the 148 participants, 20 tested positive (13.5%), with another 17 testing likely positive (11.5%). These two groups were combined to estimate overall seroprevalence at 25%. A total of 51 participants’ samples returned results in the equivocal range (34.5%), and 60 (40.5%) had %EV < 15.9, or a negative result. Of the 12 clinically diagnosed participants, only one (8.4%) returned a %EV in the negative range; this was a presumptive case (diagnosed by symptoms only) 5 years before blood draw. A total of five clinically diagnosed participants had results in the equivocal range (41.7%), and six (50%) returned %EV > 48 (likely positive and positive). Of participants self-reporting exposure or infection (*N* = 21), five (23.8%) tested positive, three (14.3%) tested likely positive, seven (33.3%) tested equivocal, and six (28.6%) tested negative. Of participants self-reporting no exposure or infection (*N* = 115), 11 (9.6%) tested positive, 12 (10.4%) tested likely positive, 39 (34%) tested equivocal, and 53 (46%) tested negative. The self-reported unexposed/uninfected population (*N* = 115) showed a mean %EV of ∼26.4.

**Figure 2.**
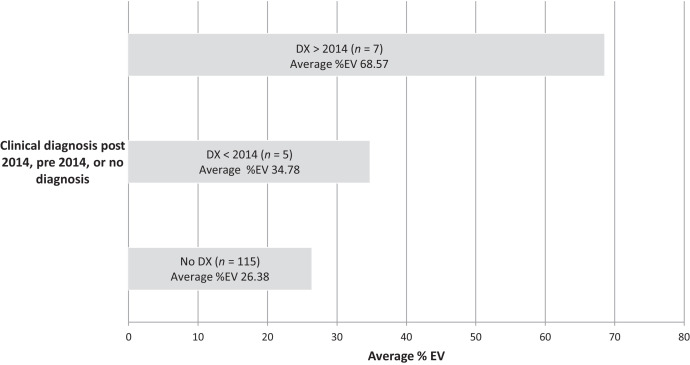
Average %EV of Hawaii 31 kDa ELISA. Of those clinically diagnosed (*n* = 12), %EV was lower (34.8) among those infected before 2014 (*n* = 5) compared with those infected post-2014 (*n* = 7) (%EV 68.6). Participants reporting no diagnoses (*n* = 115) showed the lowest %EV (26.4). DX = diagnoses; EV = ELISA value.

To evaluate temporal effects on %EV, the results of the samples of the clinically diagnosed participants were sorted as either pre-2014 or post-2014 diagnoses; the results of that comparison can be seen in Figure 2. The mean %EV of post-2014 clinically diagnosed participants was ∼68.6% (*N* = 7), whereas those diagnosed before 2014 (*N* = 5) had a much lower mean %EV of ∼34.8; however, this was not statistically significant (*P* = 0.066). Those reporting no diagnosis had a much lower average %EV of 26.4.

ELISA results using the isogeographic 31-kDa antigen were tabulated alongside the crude Hawaii antigen ELISA and the Thailand 31-kDa dot-blot results previously reported^64^ (Table 3). Those testing ELISA positive using the Hawaii isolate of the 31-kDa antigens (25%) in this study are comparable to those previously reported positive by Hawaii crude antigen ELISA (22%) and the 31-kDa dot blot using the Thailand antigen (30%).^64^ In Table 4, the Hawaii 31 kDa %EV results are categorized as positive or negative Thailand 31-kDa dot-blot results.^64^ As might be expected, the dot-blot positive sample percentage decreases in parallel with the %EV, and inversely, the dot-blot negative sample percentage increases as the %EV decreases.

**Table 3:** Comparison of Hawaii crude antigen ELISA and Thailand 31-k Da dot-blot results^64^ with Hawaii 31-kDa ELISA results by self-reported diagnosis

Diagnosis status	Total	Definitively diagnosed	Positive by self-diagnosis	Negative by self-diagnosis
Hawaii *A. cantonensis* crude antigen	*N* = 435	*N* = 15	*N* = 105	*N* = 315
Positive	97 (22%)	10 (67%)	24 (23%)	63 (20%)
Equivocal	198 (46%)	5 (33%)	46 (44%)	147 (47%)
Negative	140 (32%)	0 (0%)	35 (33%)	105 (33%)
31-kDa dot-blot antigen	*N* = 186	*N* = 15	*N* = 45	*N* = 126
Positive	56 (30%)	7 (47%)	13 (29%)	36 (29%)
Negative	130 (70%)	8 (53%)	32 (71%)	90 (71%)
Hawaii 31 kDa *A. cantonensis* antigen, ELISA	*N* = 148	*N* = 12	*N* = 21	*N* = 115
Positive	37 (25%)	6 (50%)	8 (38%)	23 (20%)
Equivocal	51 (34.5%)	5 (42.6%)	7 (33.3%)	39 (34%)
Negative	60 (40.5%)	1 (8.4%)*	6 (28%)	53 (46%)

*A. cantonensis* = *Angiostrongylus cantonensis.*

* This case was presumptively diagnosed in 2010, 5 years before sample draw.

**Table 4:** Comparison of the results of this cohort by Hawaii 31-kDa ELISA (categorized by % EV) based on positive or negative results of the Thailand 31-kDa dot-blot^64^

Diagnosis status	Positive > 70.1 %EV	Likely positive 48.1–69.9 %EV	Equivocal 16.0–47.9 %EV	Negative 0–15.9 %EV
Dot-blot positive	12 (63%)	5 (42%)	6 (23%)	1 (15%)
Dot-blot negative	7 (37%)	8 (58%)	20 (77%)	6 (85%)
Total	19	12	26	7

EV = ELISA value. Among dot-blot positive samples, percentages decrease in parallel with %EV, whereas an inverse relationship is seen among dot-blot negative samples where the percentage increases as %EV decreases.

## DISCUSSION

Eosinophilic meningitis can be caused by a number of parasites, but most cases worldwide are attributable to *A. cantonensis*.^66^ DNA diagnosis is currently achieved with a species-specific assay based on the ITS1 developed by the CDC.^49^ Before the development of ITS1 PCR, retrieval and subsequent morphological identification of nematodes from spinal fluid was the only way to verify that the etiologic agent was indeed *A. cantonensis*. This finding was both rarely noted and time-consuming. Advancements in DNA diagnosis are an improvement on the classical parasitological diagnosis.^47^^,^^49^ However, current PCR assays on CSF are still somewhat hit or miss; ITS1 was found to have an ∼33% failure rate in CSF^49^ even in progressed disease, whereas the recently developed Acan3990 assay based on repeated elements in the *A. cantonensis* genome was determined to be up to 1,000× more sensitive than the ITS assay.^47^

If anthelminthic treatment is withheld until diagnosis by DNA detection, the result may be life-threatening disease and/or long-term neurological sequelae.^27^^,^^28^^,^^45^ Treatment with anthelminthics in the 7- to 21-day window post–symptom onset is positively corroborated by experimental studies^67^^–^^70^ and by epidemiology^71^^,^^72^ and is expressly indicated as prophylaxis when known or suspected exposure to a slug or snail occurs in certain endemic regions.^61^^,^^73^ A recent study^62^ has demonstrated the in vivo efficacy of pyrantel pamoate (OTC pinworm medicine) as a postexposure prophylactic and has been included in the clinical treatment protocols at Hilo Medical Center, Hilo, HI. From rat studies, we know that after ingestion, the parasite moves quickly from the digestive tract into the bloodstream.^65^^,^^74^^–^^76^ In the definitive host, the L3 parasites can reach the peripheral blood by 53 minutes postchallenge^65^ and the central nervous system in < 17 hours.^74^^,^^75^ L3 exposure by other routes may take even less time if the bloodstream is accessed directly through abraded skin, unabraded skin, and/or mucous membranes.^76^

Enzyme-linked immunoassays (EIA) are among the most common in vitro diagnostic tools used in clinical practice.^77^ ELISA and other forms of EIA are routinely and reliably used in the diagnosis of parasitic infections such as cysticercosis, filariasis, gnathostomiasis, hydatidosis, schistosomiasis, strongyloidiasis, toxocariasis, toxoplasmosis, and trichinellosis.^78^ Crude antigen preparation of *A. cantonensis* has shown some cross-reactivity with other parasitic antigens,^52^ whereas tests using the 31-kDa glycoproteins of *A. cantonensis* have shown specificity and sensitivity of 100%.^51^ Some studies hypothesize that native antigen may be more effective at detecting patent infection in certain non-endemic areas^49^^,^^52^; others attribute the dearth of serological testing to the lack of availability of native antigen^51^^,^^55^^,^^79^^,^^80^ or lack of availability of the test itself.^63^^,^^80^ Specifically, the lack of native 31-kDa antigen has been noted as a potential barrier to serology in areas where the Thai isolate has been tested.^52^ Harvesting of local nematodes may aid in the production of a location-specific antigen.^49^

To our knowledge, this is the first study to purify 31-kDa native antigen successfully from *A. cantonensis* nematodes in the United States. Attempts to express the 31-kDa glycoproteins in host cells through cloning and antigen production have resulted in reduced specificity, likely due to complex eukaryotic carbohydrate incorporation during protein expression and the irreproducibility of protein folding.^81^ The cloned and expressed galectin-2 protein of 31 kDa also showed reduced sensitivity and specificity indicating some degree of cross-reactivity.^53^^,^^54^ A study in Tenerife rats^82^ showed an ELISA using isogeographic 31-kDa antigen (isolated from Canary Island rats) correlated well (*R*^2^ = 0.954, *P* < 0.05) with adult *A. cantonensis* found on dissection; this study also found that sera from rats infected with other parasites did not react to the 31-kDa antigen. It has also been previously reported that reducing agents such as dithiothreitol used in antigen isolation may increase cross-reactivity^52^; our antigen isolation procedure did not use reducing agents.

Our study used one time point of blood sampling, and the most recent clinically diagnosed participant tested was already several months postdiagnosis at the time of sample collection. Antibodies to Thailand 31-kDa antigen in sera were found 4 to 67 days post–symptom onset,^49^ indicating that antibodies may persist for some time. Our finding of higher OD (%EV) in recently clinically diagnosed participants compared with those with older diagnoses supports this premise; additionally, both clinically diagnosed groups showed higher OD (%EV) than the clinically undiagnosed portion of the population tested.

Although some of the higher %EV scores we found in our cohort do not seem to have a relationship with the participants’ original self-reported disease status, these high %EVs may be explained by previous undetected and/or repeated exposure. Paratenic hosts, skin transmission, and contaminated food and water have all been described or documented as routes of transmission^2^^,^^30^^,^^83^^–^^85^ and may be contributors to the high %EVs found in persons not reporting diagnosis or symptoms. We also cannot rule out that some of these participants may have become symptomatic or been diagnosed with angiostrongyliasis only after the samples were collected for this study, unbeknownst to us. For example, one participant who initially self-reported no infection/exposure later reported that a worm was removed from their eye chamber before the previous study.^64^ The participant self-reported that the excised nematode was identified as *A. cantonensis.* This participant’s serum returned positive results in this study and by both measures used in the previous pilot study. This case was not recognized by the Hawaii Department of Health as a definitively diagnosed *A. cantonensis* case due to its failure to meet the diagnostic standard (detection of DNA in CSF). Additionally, mild or asymptomatic infection may influence serological results, and individual immunoprofiles may react variably, as seen in testing for COVID-19 antibodies.^86^ Prospective, longitudinal studies could help delineate the course of antibody production.

Hawaii is a tourist destination that receives almost 10 times its population in visitors annually according to the Hawaii Department of Business, Economic Development and Tourism. According to Hawaii Department of Health data, there were nine cases of *A. cantonensis* diagnosed in 2019, of which three were in visitors. Eight cases were reported to have been acquired on Hawaii Island and one case was attributed to the island of Kauai. As an epidemiological tool, using the 31-kDa ELISA in prevalence testing has the potential to pinpoint hotspots of exposure within the hotspot of East Hawaii, which could lead to targeted intermediate and definitive host control, as well as localized educational initiatives to increase awareness in highly endemic areas with the intent to decrease incidence of disease.

Until seroconversion rates can be established through prospective studies describing the rise and fall of antibodies in active infection, indirect ELISA or dot blots with Hawaii 31-kDa antigen may be useful in diagnosis of angiostrongyliasis and neuroangiostrongyliasis. Seroconversion studies are lacking, cases are rare, and data are generally retrospective. Future studies using the 31-kDa proteins isolated in Hawaii may help elucidate the time between exposure and seroconversion. Epidemiological surveys and clinical diagnoses in Hawaii will no doubt be improved with serological testing using this isogeographic 31-kDa antigen.
